# Models of Telehealth Service Delivery in Adults With Spinal Cord Injuries: Scoping Review

**DOI:** 10.2196/41186

**Published:** 2023-06-29

**Authors:** Shaghayegh Mirbaha, Ashley Morgan, Ada Tang, Jenna Smith-Turchyn, Julie Richardson

**Affiliations:** 1 School of Rehabilitation Science McMaster University Hamilton, ON Canada

**Keywords:** community-dwelling adults with spinal cord injury, models of telehealth services, remotely delivery of health care, SCI, scoping review, spinal cord injury, telehealth, telemedicine, telerehabilitation, web-based care

## Abstract

**Background:**

In Canada, approximately 86,000 people live with spinal cord injury (SCI), and there are an estimated 3675 new cases of traumatic or nontraumatic etiology per year. Most people with SCI will experience secondary health complications, such as urinary and bowel issues, pain syndrome, pressure ulcers, and psychological disorders, resulting in severe chronic multimorbidity. Moreover, people with SCI may face barriers in accessing health care services, such as primary care physicians’ expert knowledge regarding secondary complications related to SCI. Telehealth, defined as the delivery of information and health-related services through telecommunication technologies, may help address some of the barriers, and indeed, the present global COVID-19 pandemic has emphasized the importance of integration of telehealth in health care systems. As a result of this crisis, health care providers have increased the usage of telehealth services, providing health services to individuals in need of community-based supportive care. However, the evidence on models of telehealth service delivery for adults with SCI has not been previously synthesized.

**Objective:**

The purpose of this scoping review was to identify, describe, and compare models of telehealth services for community-dwelling adults with SCI.

**Methods:**

This scoping review follows the PRISMA-ScR (Preferred Reporting Items for Systematic Reviews and Meta-Analyses Extension for Scoping Reviews) guidelines. Studies published between 1990 and December 31, 2022, were identified by searching the Ovid MEDLINE, Ovid Embase, Ovid PsycINFO, Web of Science, and CINAHL databases. Papers with specified inclusion criteria were screened by 2 investigators. Included articles focused on identifying, implementing, or evaluating telehealth interventions, including primary health care services and self-management services delivered in the community and home-based settings. One investigator performed a full-text review of each article, and data extraction included (1) study characteristics; (2) participant characteristics; (3) key characteristics of the interventions, programs, and services; and (4) outcome measures and results.

**Results:**

A total of 61 articles reported telehealth services used for preventing, managing, or treating the most common secondary complications and consequences of SCI, including chronic pain, low physical activity, pressure ulcers, and psychosocial dysfunction. Where evidence exists, improvements in community participation, physical activity, and reduction in chronic pain, pressure ulcers, etc, following SCI were demonstrated.

**Conclusions:**

Telehealth may offer an efficient and effective option for health service delivery for community-dwelling individuals with SCI, ensuring continuity of rehabilitation, follow-up after hospital discharge, and early detection, management, or treatment of potential secondary complications following SCI. We recommend that the stakeholders involved with patients with SCI consider the uptake of hybridized (blend of web-based and in-person) health care delivery models to optimize the care continuum and self-management of SCI-related care. The findings of this scoping review may be used to inform policy makers, health care professionals, and stakeholders engaged in establishing web-based clinics for individuals with SCI.

## Introduction

### Background

In Canada, approximately 86,000 people live with spinal cord injury (SCI), with an additional 3675 new cases each year [1]. SCI leads to a full or partial loss of sensory, motor, and autonomic function that can be severe and often life-threatening [2]. SCI is commonly classified by distinct characteristics of the injury, such as the mechanism of injury (traumatic or nontraumatic), type of injury (complete vs incomplete), level of neurological spinal injury (cervical-, thoracic-, lumbar-, and sacral-level injuries), and the severity of injury widely rated by American Spinal Injury Association Impairment Scale [3].

SCI may result in severe chronic morbidity from secondary complications that can be chronic in nature [4] and include respiratory concerns, urinary and bowel issues, chronic pain, pressure ulcers, psychosocial complications, bone fracture, and osteoporosis [4]. These may lead to decreased functional independence, health-related quality of life, and community participation, as well as increased rates of hospitalization and loss of employability [5]. Thus, despite the relatively low incidence and prevalence rates of SCI, the associated health conditions contribute to a significant economic impact and financial burden for the patients and their caregivers [6]. In Canada, the net lifetime cost of a person with SCI is estimated to vary between CAD $1.5-$3 million (US $1.10-2.21) [6], and evidence suggests that the direct health care cost of people with SCI is 8 times that of their non–SCI age-matched peers [6]. Therefore, strategies that focus on prevention, early detection, and management of the sequelae of SCI are critical to improve functional level and quality of life of community-dwelling adults with SCI [5,7,8]. Improved self-management skills, rehabilitation, and access to proactive multidisciplinary health care teams are needed to reduce the morbidity and mortality rates associated with chronic conditions in patients with SCI, as well as alleviate the economic burden [9,10].

People with SCI may face several barriers in accessing health care services, including lack of transportation, physical obstacles, lack of preventative health screenings, and primary care physician’s expert knowledge regarding secondary complications and preventative care issues related to SCI [11,12]. Research suggests that adequate access to primary health care services may reduce the risk of developing long-term secondary complications associated with SCI as well as chronic illnesses [11,12]. However, a scoping review by McColl et al [12] found that despite patients with SCI being significantly dependent on primary care, their various medical needs, especially those related to rehabilitation services, are poorly met [12].

Telehealth is a strategy that may help to fill unmet care needs among individuals with SCI. Telehealth is defined as the delivery of information and health-related services through telecommunication technologies [13]. Moreover, the present global COVID-19 pandemic has further emphasized the importance of telehealth, facilitating the continuation of primary health care services for patients who need supportive care [14]. Telerehabilitation is a growing application of telehealth that involves the remote delivery of rehabilitation services, including training, education, self-management, compensatory strategies, and monitoring, to patients with impairments and disabilities [14]. Literature suggests that several telerehabilitation and telehealth programs exist, particularly for patients with SCI, ensuring continuity of rehabilitation and follow-up after discharge to prevent secondary complications [11]. As telehealth is increasingly used to help with the delivery and follow-up of health care services, especially during the present global COVID-19 pandemic, there remains a need to determine the status of telehealth for community-dwelling adults with SCI.

### Objectives

With the increasing use of telehealth for delivery and follow-up of health care services for patients with SCI, a scoping review was conducted to systematically review and identify the gaps in the literature. The scoping review is an emerging literature synthesis method that emphasizes covering broad, comprehensive objectives and research questions rather than a particular standard of evidence [15]. This methodological approach is particularly useful when addressing a concept with emerging evidence that applies to telehealth research targeting the SCI population. The purpose of this scoping review was to identify, describe, and compare models of remotely delivered rehabilitative interventions and health services for patients with SCI living in the community. The research question guiding this scoping review was “What models of telehealth and telerehabilitation services are available to community-dwelling adults with SCIs?” Through this review, we identified (1) characteristics (eg, types of telerehabilitation services provided, format, delivery, intensity, frequency, duration, technology component, underlying framework or theories, etc) of distinct telehealth interventions and services used to prevent, treat, and manage secondary complications of SCI; (2) characteristics of the target population (eg, age, sex, level of injury, time since injury, related secondary complications, etc); (3) characteristics of studies conducted (eg, qualitative, quantitative, mixed methods, systematic reviews, scoping reviews, etc); and (4) outcomes examined. The findings of this scoping review would be tailored toward informing the key stakeholders who are involved in establishing a web-based clinic for community-dwelling adults with SCI in a postpandemic world in Ontario, Canada, which is the ultimate goal of this project.

## Methods

This scoping review was performed in accordance with the framework from Arksey and O’Malley [15]. The PRISMA-ScR (Preferred Reporting Items for Systematic Reviews and Meta-Analyses Extension for Scoping Reviews) was also used to guide the reporting of this scoping review (see Multimedia Appendix 1 for completed PRISMA-ScR checklist) [16].

### Eligibility Criteria

To be included in this review, articles needed to focus on the identification, implementation, and evaluation of telehealth and telerehabilitation interventions, including primary health care services and self-management services delivered in the community and home-based settings. Refer to Textbox 1 for details on the inclusion and exclusion criteria.

**Summary of inclusion and exclusion criteria.**

**Inclusion criteria:**

- Qualitative, quantitative, mixed methods studies, sources of expert opinion, textual and narrative data, and reviews
- Community-dwelling adults with spinal cord injury (SCI)
- Adults aged 18 years or older
- Studies conducted in high-income countries as the results of this research were specifically tailored to the Canadian SCI community based on the North American health care system.
- Published between 1990 and December 2022
- Articles reported in English

**Exclusion criteria:**

- Abstracts only, conferences and posters, study protocols without the published full-text article, editorial letters, and 1-page commentaries
- Person with SCI who is not living within the community (ie, being treated as an inpatient)
- Person who is younger than 18 years
- Studies conducted in middle- or low-income countries
- Published before 1990 as the majority of research on the topic of telehealth and thus the use of telecommunication technologies to aid the delivery and follow-up of health care services occurred after this date.
- Non-English studies

### Search Strategy and Information Sources

A comprehensive search strategy was performed by the primary investigator (SM) to include all relevant literature published between 1990 and December 31, 2022, using the following databases: Ovid MEDLINE (1946-Dec 2022), Ovid Embase (1974-Dec 2022), Ovid PsycINFO (1806-Dec 2022), Web of Science, and CINAHL (1985-Dec 2022). Initially, the review was planned to consider the literature for 3 separate themes. Theme 1: primary care services delivered remotely to patients with SCI; theme 2: telerehabilitation services delivered remotely to patients with SCI; and theme 3: self-management interventions delivered remotely to patients with SCI. However, following consultation with a Medical Librarian in Health Sciences at McMaster University, the authors ran 1 overarching search using medical subject headings and text words related to web-based care and SCIs. Investigators reviewed the final search results to ensure all the initial theme-based articles were included in the overarching search result that was planned to be used, followed by the final decision to select the most appropriate search strategy. The final search strategies can be found in Multimedia Appendix 2. A manual search of the reference lists of recent studies was conducted to ensure the inclusion of all relevant articles in the scoping review. Previously published systematic, narrative, and scoping reviews of related topics were reviewed for related results to ensure all relevant references were included in this review.

### Study Selection

Eligible articles were identified using a 3-step process. In step 1, one reviewer (SM) collected the search results and then removed the duplicates. In the second step, 2 reviewers (SM and AM) independently reviewed the titles and abstracts of the identified articles that appeared to meet the inclusion criteria. In step 3, the same reviewers evaluated the full texts of the remaining publications identified by our searches to include potentially relevant publications. Covidence software was used to support and synthesize the process of scoping review production [17]. Discrepancies were resolved through discussion between the reviewers. If reviewers failed to reach a consensus, a third expert reviewer (JR) was consulted for the final decision about inclusion.

### Data Extraction

Data from eligible studies were extracted using data extraction forms developed by the research team (refer to the Multimedia Appendix 3). Two separate data extraction tools were designed for qualitative and quantitative studies. Information extracted from studies included study characteristics (eg, year of publication, country of study, purpose of the study, and study design), participant characteristics (eg, age, sex, time since injury, detail of SCI, and secondary complications related to SCI), key characteristics of the interventions, programs, and services (eg, description, the format of delivery, facilitator, duration, frequency, intensity, underlying theories for the intervention, types of intervention, and the type of technology modality), outcome measures, and results. The extracted results were examined to determine trends in telehealth service components and characteristics among community-dwelling adults with SCI. For qualitative studies, themes and findings were collated by the lead reviewer (SM).

### Data Synthesis

In this review, the extracted results were classified under the main categories for which the services were delivered. In this study, the extracted data were synthesized using both numerical and descriptive analysis, providing both a narrative description of the quantity of articles that address particular issues and a descriptive overview of the types of evidence available on this topic of interest.

## Results

### Source of Evidence

**Figure 1:**
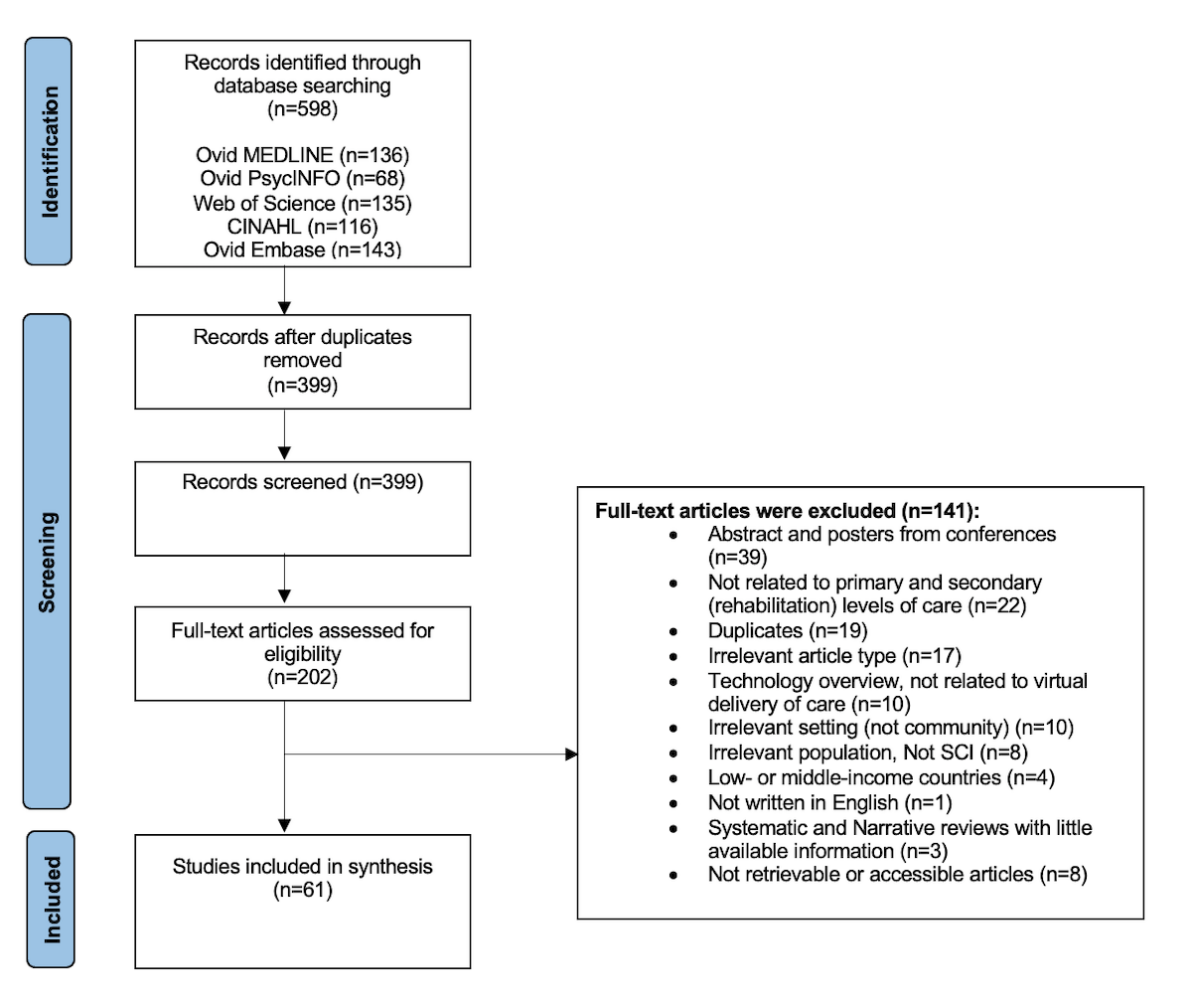
PRISMA (Preferred Reporting Items for Systematic Reviews and Meta-Analyses) flowchart. SCI: spinal cord injury.

Initially, 598 studies were imported into the Covidence software. After duplicates were removed, 399 citations were detected from searches of electronic databases and article references. After the title and abstract screening, 202 articles were included for the full-text review process. Of these 202 articles, 141 were excluded (see Figure 1 for reasons), and 61 studies met the inclusion criteria. Data extraction was completed for the remaining 61 studies, which were considered eligible for this review. The PRISMA flowchart is shown in Figure 1.

### Study Characteristics

A descriptive summary for each experimental and nonexperimental study is included in Multimedia Appendices 4 and 5, respectively [7,11,13,14,18-75].

The included papers consisted of 14 qualitative studies, 29 quantitative studies conducted in one-on-one format, 4 mixed method studies, 4 systematic reviews, 2 scoping reviews, 5 conceptual reviews, 2 narrative reviews, and 1 meta-analysis. These studies were conducted in 9 countries: the United States (n=32), Canada (n=13), Australia (n=7), Italy (n=2), South Korea (n=2), the United Kingdom (n=1), the Netherlands (n=1), Sweden (n=1), and Norway (n=2). The first article on telehealth for SCI community-dwelling adults with SCI was published in 1997, with an upward trend starting in 2015 with 51 related articles published on this topic in the last 18 years.

### Participant Characteristics

The mean sample size of the included articles was relatively small, with an average of 59 participants, but ranged from 1 to 15,028 across quantitative (n=29) and qualitative (n=13) studies that specified sample size. The age of participants ranged from 18 to 85 years.

### SCI Type

Approximately, only half of the included studies (n=34) reported the injury characteristics of the participants. The type of SCI was indicated by 2 studies, where participants had both traumatic and nontraumatic injuries [20,21]. Injury levels (ie, cervical, thoracic, and lumbar spine injuries), neurologic completeness, and paralysis (paraplegia and tetraplegia) varied among the participants of the included studies, indicating heterogeneous nature of participants under investigation.

### Outcomes

#### Physical Activity and Leisure Time Physical Activity Motivation and Participation

A total of 14 articles focused on improving physical activity (PA) and leisure time physical activity (LTPA) participation, motivation, and exercise endurance [18,20,21,23-25,27,​^36^,^43^,^45^-^47^,^49^,^50^]. There were 11 articles that quantitatively assessed the effectiveness of interventions and services to improve motivation and participation in PA and LTPA (Multimedia Appendix 4) [18,20,21,23-25,27,36,43,45,46]. Of the 11 studies, 3 (27%) used telephone counseling sessions [21,24,43], 4 (36%) studies applied web-based applications or platforms providing contents (ie, modules, homework, videos, and email or phone support from a provider, coach, or peer) [18,23,45,46], 1 (9%) study employed wearable sensors connected to a mobile phone, transferring collected data to a server [25], and the remaining 3 (27%) studies used videoconferencing software (video-telehealth) as a means of delivering home-based exercise interventions [20,27,36]. The duration of the interventions ranged between 2 and 6 months. A total of 9 (82%) [18,21,23-25,27,36,43,45] of the 11 studies described the planned frequency of contact with the provider, ranging from once a week to once a month, while only 1 (9%) study did not report the frequency of peer and provider interaction [46]. All 11 studies provided a combination of services aimed at monitoring, providing feedback, and providing motivational and informational support to the target population. Overall, 6 studies [18,21,27,43,46,47] indicated that an exercise, PA, or LTPA counselor provided the intervention, whereas 2 studies [43,46] reported registered kinesiologists who were trained in motivational interviewing techniques and behavioral change theories and facilitated the service provision. In contrast, 4 studies [23,24,49] reported that an experienced physiotherapist monitored the self-regulatory interventions. Only 1 study [36] reported that the telehealth coordinator monitored the training of the participants involved in the videoconference program. The outcomes most frequently used for measuring PA and LTPA were the 7-day Self-report LTPA Questionnaire and World Health Organization Quality of Life BREF Scale. In most of the studies (n=10) [18,21-23,25,27,36,43,45,46], the interventions had favorable outcomes, elevating the participant’s engagement in LTPA and PA levels. One pilot randomized controlled trial (RCT) [46] was designed to pilot-test a telehealth intervention, grounded in the self-determination theory, to improve PA and quality of life among patients with SCI. The results of this pilot RCT [46] showed that telephone counseling represents a promising way to decrease health care use during the first year following SCI. Another pilot RCT [24] was conducted to test the effectiveness and feasibility of telephone counseling interventions compared to standard care of increasing physical fitness and reducing medical complications in patients with SCI, indicating no improvement in LTPA and PA levels but decreased depressive symptoms [24].

#### Chronic Pain

A total of 2 quantitative pre-post studies [32,38] and 1 qualitative study [52] focused on self-management strategies for reducing chronic pain in adults with SCI (see Multimedia Appendices 4 and 5). Of the 2 quantitative studies, 1 used a web-based chronic pain program, while the other used videoconferencing software (video-telehealth) to educate participants on self-management skills and exercise training to reduce pain, respectively [32,38]. In both studies, an experienced physiotherapist monitored and contacted participants if needed [32,38]. All studies reported the duration of the intervention, which ranged between 8 weeks and 6 months. By contrast, only 2 studies noted the frequency of contact, which ranged from 3 times per week to 2 calls per month [32,52]. The 2 quantitative studies reported on the measures used to assess chronic pain, including the Pain Disability Index [38], Pain Self-efficacy Questionnaire [38], and the Shoulder Rating Questionnaire [32], which measures upper extremity function and pain intensity. The 2 quantitative studies demonstrated reduced pain and improved pain-related disability among adults with SCI [32,38]. Both chronic pain and psychosocial outcomes were targeted by 1 study, resulting in a reduction in pain and depression and improved life satisfaction [38]. The qualitative study, which explored peer health coaches’ role as provider, highlighted the important role of peer health coaches in promoting chronic pain self-management strategies among peers with SCI [52].

#### Pressure Ulcers, Sores, Injuries, and Skin or Wound Care

Overall, 11 articles specifically focused on prevention, treatment, or self-management of pressure ulcers and wound care (see Multimedia Appendices 4 and 5) [30,34,37,53-59,72]. Out of the 11 articles, 4 (36%) studies used a videophone (Picasso AT&T) modality to transmit still images and audio over a standard telephone line and to hold audio-video consultation sessions with the SCI population [30,37,53,54]. All 4 studies provided monitoring, wound and pressure ulcers management services, and informational support. The frequency of contact with providers varied, with an average of 7 visits per month. The duration of interventions varied from 6 weeks to 12 weeks. The only outcome measure used to assess pressure ulcers was the Pressure Ulcer Scale for Healing tool version 3.0 [53]. All the interventions demonstrated favorable results in successfully managing pressure injuries and wounds through telehealth. One recent RCT [72] on health-related quality of life and satisfaction of patients with SCI and pressure injuries receiving real-time multidisciplinary videoconference consultations (video-telehealth) found videoconference-based care to be a safe and efficient way of managing pressure injuries, particularly for those individuals requiring long-term follow-up care and living far from the wound specialists. A total of 2 systematic reviews [55,56] and 1 scoping review [57] identified evidence to inform the development of telehealth techniques used to prevent, treat, and self-manage pressure ulcers in patients with SCI following discharge. One qualitative study that used semistructured interviews [34] and 1 modeled analysis [58] reported on patients’ experience using an educational mobile app for pressure ulcer prevention and evaluated costs and savings associated with telehealth services for preventing and treating pressure ulcers, respectively. The results of the modeled analysis found that telehealth services were less expensive than standard care when low-cost technology was used but more expensive in cases where high-cost interactive devices were applied in patients’ home settings [58].

#### Psychosocial Function

A total of 8 articles focused on the effects of telehealth services in reducing psychosocial problems such as depression, anxiety, life satisfaction, community participation, and reintegration (see Multimedia Appendices 4 and 5) [13,28,40,41,51,60-62]. Telehealth modalities and delivery methods included telephone counseling through simple telephone lines [13,61,62], mobile apps [28], video-based counseling sessions using videoconferencing software (video-telehealth) [13], and web-based applications on any devices accessing internet networks [40,41,51]. In 2 studies involving telephone and video counseling sessions, interventions were provided by a trained nurse and peers who have lived experience with SCI trained in motivational interviewing techniques [13,28]. In 2 studies, patients used the web-based interventions independently without facilitator involvement or supervision [40,41]. The most frequent measures used to assess psychosocial functioning were Depression, Anxiety, and Stress Scale–Short Form [40,41], the Quality of Well-Being Scale [13,41], Craig Handicap Assessment and Reporting Technique Short Form 10 assessing community participation [26,29,41,42], and Patient Health Questionnaire-9 measuring depression severity [28]. The results from the 4 studies were inconsistent in the effects of psychosocial treatments on depressive symptoms, life satisfaction, and quality of life. Improvements in depressive symptoms were demonstrated in 2 studies, such as improved sleep and psychomotor symptoms, positive appetite changes, and increased energy [40,41]. In contrast, 2 studies, including an RCT [28], exploring the use of the mobile app (iMHere) on psychosocial outcomes, showed no improvement in psychosocial and health-related quality of life outcomes [13,28]. Overall, 2 qualitative studies reported on models of service delivery supporting community reintegration and efficiency of psychological interventions delivered by telephone on emotional outcomes [51,60]. One meta-analysis and 1 systematic review evaluated the impact of remotely delivered psychological interventions on the psychological functioning of adults with SCI [61,62]. The systematic review [62] results indicated telecounseling enhanced management of common comorbidities following SCI, including pain and sleep difficulties. The meta-analysis [61] results demonstrated significant enhancement in coping skills and strategies, depression, and community reintegration following SCI. Ten studies used a cognitive behavior change theory and psychological principle in addressing cognitive behavioral aspects of psychosocial conditions following SCI [20,21,26,38,40,41,43,46,50,51].

#### Addressing Multiple Secondary Complications

Twenty studies focused on multiple secondary complications, including pressure ulcers or wound care, urinary tract infections, a range of psychosocial problems, and community participation, instead of addressing a single complication (see Multimedia Appendices 4 and 5) [7,11,14,22,26,29,31,39,42,44,48,63-66,​^69^,^70^,^73^-^75^]. From the 7 studies [7,26,29,31,42,44] which evaluated interventions for multiple secondary complications, telehealth delivery included videoconferencing software (video-telehealth) (n=2) [31,44], web-based application (n=1) [39], mobile apps (n=1) [42], telephone (n=1) [29], and automated calls using interactive voice response system (n=2) [7,26]. The duration of the interventions ranged from 4 months to 9 months. Only 3 studies reported the frequency of interventions, ranging from once a week to biweekly interactions [29,31,44]. The multicomponent interventions were either provided by registered nurses (n=1), physiotherapists (n=2), occupational therapists (n=1), SCI specialists (n=1), or primary care physicians (n=1). Study results varied from no improvement to significant positive outcomes. Narrative reviews (n=2) [63,65], a systematic review (n=1) [66], conceptual review (n=1) [64], and qualitative studies (n=2) [11,48] reported the use of telehealth for patients with SCI who had multiple secondary complications. The patient-provider’s perspective about the potential effects of telehealth services regarding the occurrence of secondary complications as well as higher levels of engagement with web-based peer support components, respectively, was described in the qualitative studies [11,48]. In a recent mixed methods study comparing videoconferencing to in-person peer support, most participants felt socially connected with web-based peer support [73]. Overall, 2 conceptual reviews [14,69] and 2 qualitative studies [74,75] discussed how, in the time of COVID-19, telerehabilitation services addressing multiple secondary complications maintained patient-provider interaction and access to essential health care services. These services may otherwise have been interrupted by physical isolation and social distancing regulations in place during the global COVID-19 pandemic [14,69,74,75]. A recent qualitative study [75] explored experiences of persons with SCI with tele-SCI services during the COVID-19 pandemic in British Columbia, Canada, suggesting the presence of benefits from blended models of health care delivery (combination of web-based and in-person care) for the SCI community in a postpandemic world. This qualitative study also explored the expected benefits (ie, increased accessibility and convenience) and challenges (ie, poor infrastructure and limitations in hands-on physical examination performance) of telehealth applications from the perspective of the SCI community [75]. In addition, 1 cross-sectional descriptive study [22], 1 qualitative study [19], and 1 literature review [70] investigated the models of telehealth use provided by United States Army Veterans programs, particularly veterans with SCI. These 3 studies mainly discussed the important role of leadership support merged with telehealth technologies allowing for follow-up of long-term self-management of patients with SCI [19,22,70].

#### Oral Health

A total of 2 quantitative studies reported participants’ satisfaction with, adaptability of, and user friendliness of home oral care telecare programs, leading to improved oral and gingival health in patients with SCI [33,35]. Both studies used videoconferencing software (video-telehealth) requiring high-speed internet connections as remote modalities facilitated by occupational therapists. These studies reported factors enabling the implementation of telehealth services to address oral care and described ways to integrate tele-oral care services into routine clinical practice [33,35].

## Discussion

### Principal Findings

This scoping review mapped the literature about the telehealth services provided to community-dwelling adults with SCI. In this review, the extracted results are classified under the main categories for which services were delivered. These included PA or LTPA motivation and participation, chronic pain, pressure ulcers or sores, skin or wound care, psychosocial dysfunction, and oral health. There were 61 articles included in this scoping review; 29 of the articles were quantitative studies, 14 qualitative studies, 4 mixed method studies, and the remainder were different review types (ie, narrative reviews, scoping reviews, systematic reviews, and conceptual reviews), mainly to explore how the existing telehealth services focus on self-management of different SCI-related secondary complications. The results of this paper also highlighted the importance of telerehabilitation services in the time of COVID-19 to increase access to health care services for community-dwelling adults with SCI.

There was inconsistency in reporting the demographic characteristics of participants, particularly the level and type of injury, time since injury, and the SCI-related secondary complications present at the time of participation in the included studies. In addition, most of the included articles needed to identify and describe the theories and frameworks used to construct telehealth services. In most of the included articles with human participants, sample size sufficiency reporting was often considered poor, characterized as a relatively small sample size, and discussed in the context of study limitations. Insufficient and relatively small sample sizes are known to undermine the validity and generalizability of the study results [76]. There was an upward trend in the number of publications since 2015. This upward trend may reflect an increase in the need for telehealth interventions, together with the availability, acceptability, and increased competency with telehealth technologies by health care professionals and patients with chronic neurologic conditions, particularly community-dwelling adults with SCI [2-5,7-9,11,14,17-19,21-24,26-28,30,33,​^35^,^37^,^40^-^46^,^48^-^51^,^54^,^56^,^59^,^62^,^64^,^67^,^69^]. Additionally, there was inconsistency in reporting the duration of interventions and frequency of contact with the providers.

Recent studies conducted during the COVID-19 pandemic support the need for telerehabilitation and telehealth for patients with neurological deficits such as SCI [14,68]. The most recent research indicates that community-dwelling adults with chronic conditions are becoming more engaged with using cost-efficient, easy-to-use technologies to access essential health care services, especially during the current pandemic [14,68]. For example, telehealth has been used to provide indirect contact between psychiatrists and patients, enhancing access to psychological wellness, which may be interrupted due to pandemic-related physical and social isolation [14]. Recent studies suggest hybridized health care delivery models, blending remote- or telecare with in-person care, are promising approaches to create more accessible and patient-centered care for people with SCI who live in more remote areas with limited access to in-person health care services [74,75].

A number of articles in this review evaluated the effectiveness, implementation, and use of existing telehealth services to address secondary complications. A limited number of RCTs were designed to evaluate the effectiveness and use of telehealth interventions among patients with SCI. Various formats and means of remote service delivery were used, such as web-based platforms and applications with contents such as modules, homework, educational videos, and email or phone support from a provider or facilitator, videoconferencing software, telephone counseling sessions, and automated calls using simple telephone lines. Our findings suggest greater patient-reported satisfaction levels and better interaction occur when video modalities are used rather than telephone communications during remote- or televisits [29,75]. Video-based telehealth tools improve remote care by enabling patients to see their providers, fostering therapeutic and clinical rapport, and building interpersonal relationships.

This scoping review suggests that remote, live, peer, or coach support increases patient engagement in their health, promoting health behaviors and outcomes among adults with chronic neurologic conditions. Patients with SCI appreciated being able to have face-to-face remote- or tele-interaction with their health care providers and especially highlighted the importance of designing peer-led interventions (eg, My CareMy Call peer-led interventions). They also acknowledged peer mentors’ powerful role in promoting self-management strategies to prevent secondary complications in adults with SCI. Furthermore, peer mentors are widely accepted as a source of social support while fulfilling the roles of role model, advisor, and supporter by promoting self-management skills through educating, strategizing, and more importantly, emotionally connecting and building trust with adults with SCI [52]. Telehealth expands access to care for those requiring specialty care or long-term follow-up care, allowing more frequent encounters and coaching that could facilitate patients’ active participation in their self-management of SCI-related care [42].

The findings of this review suggest that most telehealth interventions and services carried out in the home and community were combined with at least one behavioral technique and training [20,21,26,38,40,41,43,46,50,51]. Behavioral training incorporated into the interventions included goal setting, identification of barriers, problem-solving, feedback about performance, emotional support, and decision-making, which primarily resulted in the development of self-management skills and reduction or control of secondary conditions. These findings confirm the findings from the conceptual review conducted by Dobkin [50] that telerehabilitation technologies offer ways to remotely include behavioral training to self-coordinate (self-navigate and self-manage) their primary care services, resulting in reduced impairment and disability after SCI.

Additionally, the qualitative studies and conceptual reviews included in this scoping review indicate that leadership support and accepted management in health care facilities influence the implementation and maintenance of telerehabilitation use in routine practice. This finding is consistent with a study conducted by Moehr et al [71], which reported that continuous coordination and guidance by management teams lead to successful incorporation of telerehabilitation in clinical routine.

Finally, our study demonstrates that most qualitative studies reported high acceptability and satisfaction with telehealth services in patients with SCI, mainly attributed to the accessibility, convenience, and interpersonal interaction with telecare coaches and providers who have expert knowledge in preventing and treating secondary complications following SCI. The findings of this scoping review highlight the advantages of using telehealth to complement traditional in-person care when transitioning from inpatient care to community settings. Most telehealth interventions have been developed to support people with SCI during their transitions from acute rehabilitation to the community by providing psychosocial adjustments and assisting with other unexpected challenges [51,60,61]. The vast majority of articles published before and after the onset of the COVID-19 pandemic suggest that future telehealth services should be offered and delivered through blended (hybridized) models of care, enhancing access to care while still providing in-person care for those needing hands-on operations and treating and managing their SCI-related secondary conditions [74,75].

### Future Directions

The findings of our scoping review indicate that telehealth technologies are potentially effective strategies for addressing disparities in providing quality care and managing multiple health concerns for patients with SCI. The results highlighted the need for future research in the following areas: (1) increased involvement of multidisciplinary teams to facilitate interventions for managing secondary complications, as there were no intervention-based studies reported using multidisciplinary team approaches; (2) implementation of underlying theory or model to inform telehealth services supporting the intended outcomes of the intervention, as majority of the included studies lacked underlying theories or frameworks; (3) present more consistent details about population characteristics, mainly related to SCI; and (4) address gender differences in user needs and engagement with SCI.

### Limitations

First, many of the studies were conducted in the United States and Canada, where health care systems vary from other countries, limiting the generalizability of the results to a North American context. Second, this review only included studies conducted in high-income countries and thus does not reflect telehealth delivery models for patients with SCI in low-income countries. This was mainly because the research was required to be guided toward the North American health care system context to fulfill our ultimate goal of informing key stakeholders involved in establishing a web-based clinic for community-dwelling adults with SCI in Ontario, Canada. Following this study, future research needs to consider the inclusion of middle- and low-income countries to reflect telehealth delivery models for patients with SCI in an international context. Third, the mean sample size of the included articles was relatively small, affecting the generalizability of the study findings to the whole SCI population. As a result, more research with larger sample sizes is required in this field of research to improve the validity and generalizability of the study results. Last, this review was limited to English-written articles. Therefore, it is possible there may be missing studies available in other languages. Accordingly, a systematic review is needed to obtain a more accurate view on the effectiveness of telehealth models of service delivery for community-dwelling adults with SCI.

### Conclusion

This scoping review mapped the existing literature on what is known about the telehealth services provided to community-dwelling adults with SCI. Telehealth interventions and services carried out in the home and community of persons with SCI result in the development of self-management skills to reduce or control the secondary conditions following SCI. Moving forward, we expect to see a significant rise in the delivery of telehealth services through hybridized models of care, enhancing access to care while still providing in-person care for those needing hands-on care in managing SCI-related secondary conditions. Additionally, we anticipate an expansion in access to more specialty care by increasing the contact with providers who have expert knowledge in preventing and treating secondary complications following SCI. Findings from this scoping review will be used to inform the policy makers, health care professionals, and local and national stakeholders who are engaged in the planning, implementation, and funding process of establishing a web-based clinic for the SCI population.

## Appendix

Multimedia Appendix 1 Preferred Reporting Items for Systematic reviews and Meta-Analyses extension for Scoping Reviews (PRISMA-ScR) Checklist.

Multimedia Appendix 2 Final search strategies.

Multimedia Appendix 3 Developed quantitative and qualitative data extraction forms.

Multimedia Appendix 4 Complete quantitative data set.

Multimedia Appendix 5 Complete qualitative data set.

## Abbreviations

LTPA: leisure-time physical activity
PA: physical activity
PRISMA-ScR: Preferred Reporting Items for Systematic Reviews and Meta-Analyses Extension for Scoping Reviews
RCT: randomized controlled trial
SCI: spinal cord injury

## Data Availability

The data sets, filled-in data extraction forms for all the included articles, generated and analyzed during this study are available from the corresponding author on reasonable request.
