# Supplementary material for: Models of Telehealth Service Delivery in Adults With Spinal Cord Injuries: Scoping Review
Source: JMIR Rehabil Assist Technol. 2023 Jun 29;10:e41186. doi: 10.2196/41186 (PMC10365587; doi:10.2196/41186)
Supplement: Multimedia Appendix 2 [file rehab_v10i1e41186_app2.docx]

**Medline via Ovid Search Strategy**


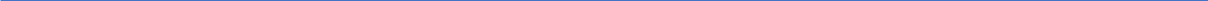


| 1 | exp Telemedicine/ |
| --- | --- |
| 2 | telemedicine.mp. |
| 3 | virtual care.mp. |

- mobile health.mp.

| 5 | telehealth.mp. |  |  |  |
| --- | --- | --- | --- | --- |
| 6 | tele-health.mp. |  |  |  |
| 7 | eHealth.mp. |  |  |  |
| 8 | mHealth.mp. |  |  |  |
| 9 | 1 or 2 or 3 or 4 or 5 or 6 or 7 or 8 | | | |
| 10 | exp Spinal Cord Injuries/ | | |  |
| 11 | spinal cord injuries.mp | | |  |

1. spinal cord contusion.mp.
2. spinal cord contusions.mp.

14 spinal cord injury.mp.

1. spinal cord laceration.mp.
2. spinal cord transection*.mp.

| 17 | spinal cord trauma*.mp. | |  |
| --- | --- | --- | --- |
| 18 | exp Paraplegia/ | |  |
| 19 | exp Quadriplegia/ | |  |
| 20 | paraplegia.mp. | |  |
| 21 | quadriplegia.mp. | |  |
| 22 | 10 or 11 or 12 or 13 or 14 or 15 or 16 or 17 or 18 or 19 or 20 or 21 | | |
| 23 | 9 and 22 |  |  |

**PsycINFO via Ovid Search Strategy**


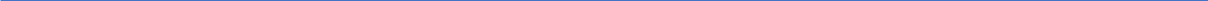


| 1 | exp Telemedicine/ |
| --- | --- |
| 2 | telemedicine.mp. |
| 3 | virtual care.mp. |

- mobile health.mp.

| 5 | telehealth.mp. |  |  |  |
| --- | --- | --- | --- | --- |
| 6 | tele-health.mp. |  |  |  |
| 7 | eHealth.mp. |  |  |  |
| 8 | mHealth.mp. |  |  |  |
| 9 | 1 or 2 or 3 or 4 or 5 or 6 or 7 or 8 | | | |
| 10 | exp Spinal Cord Injuries/ | | |  |
| 11 | spinal cord injuries.mp | | |  |

1. spinal cord contusion.mp.
2. spinal cord contusions.mp.

14 spinal cord injury.mp.

1. spinal cord laceration.mp.
2. spinal cord transection*.mp.

| 17 | spinal cord trauma*.mp. | |  |
| --- | --- | --- | --- |
| 18 | exp Paraplegia/ | |  |
| 19 | exp Quadriplegia/ | |  |
| 20 | paraplegia.mp. | |  |
| 21 | quadriplegia.mp. | |  |
| 22 | 10 or 11 or 12 or 13 or 14 or 15 or 16 or 17 or 18 or 19 or 20 or 21 | | |
| 23 | 9 and 22 |  |  |

**CINAHL Search Strategy**


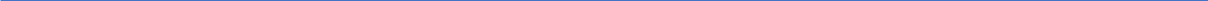


1 (MH "Telemedicine")

2 telemedicine

3 virtual care

4 mobile health

5 telehealth

6 tele-health

7 eHealth

8 mHealth

9 S1 OR S2 OR S3 OR S4 OR S5 OR S6 OR S7 OR S8

10 (MH "Spinal Cord Injuries")

11 spinal cord injuries

12 spinal cord contusion

13 spinal cord contusions

14 spinal cord injury

15 spinal cord laceration

16 spinal cord transection*

17 spinal cord trauma*

18 (MH "Paraplegia")

19 (MH "Quadriplegia")

20 paraplegia

21 quadriplegia

22 S10 OR S11 OR S12 OR S13 OR S14 OR S15 OR S16 OR S17 OR S18 OR S19 OR S20 OR S21

23 S9 AND S22

**Web of Science Search Strategy**


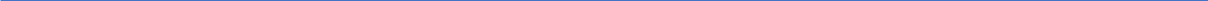


1 TOPIC: ("telemedicine")

2 TOPIC: ("virtual care")

3 TOPIC: ("mobile health")

4 TOPIC: ("tele-health")

5 TOPIC: ("eHealth")

6 TOPIC: ("mHealth")

7 #1 OR #2 OR #3 OR #4 OR #5 OR #6

8 TOPIC: ("spinal cord injury")

9 TOPIC: ("spinal cord injuries")

10 TOPIC: ("spinal cord contusion*")

11 TOPIC: ("spinal cord laceration")

12 TOPIC: ("spinal cord transection*")

13 TOPIC: ("spinal cord trauma*")

14 TOPIC: ("paraplegia")

15 TOPIC: ("quadriplegia")

16 #8 OR #9 OR #10 OR #11 OR #12 OR #13 OR #14 OR #15

17 #7 AND #16

**Embase via Ovid Search Strategy**


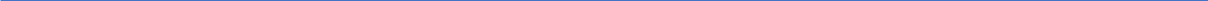


| 1 | exp Telemedicine/ |
| --- | --- |
| 2 | telemedicine.mp. |
| 3 | virtual care.mp. |

- mobile health.mp.

| 5 | telehealth.mp. |  |  |  |
| --- | --- | --- | --- | --- |
| 6 | tele-health.mp. |  |  |  |
| 7 | eHealth.mp. |  |  |  |
| 8 | mHealth.mp. |  |  |  |
| 9 | 1 or 2 or 3 or 4 or 5 or 6 or 7 or 8 | | | |
| 10 | exp Spinal Cord Injuries/ | | |  |
| 11 | spinal cord injuries.mp | | |  |

1. spinal cord contusion.mp.
2. spinal cord contusions.mp.

14 spinal cord injury.mp.

15 spinal cord laceration.mp.

16 spinal cord transection*.mp.

| 17 | spinal cord trauma*.mp. | |  |
| --- | --- | --- | --- |
| 18 | exp Paraplegia/ | |  |
| 19 | exp Quadriplegia/ | |  |
| 20 | paraplegia.mp. | |  |
| 21 | quadriplegia.mp. | |  |
| 22 | 10 or 11 or 12 or 13 or 14 or 15 or 16 or 17 or 18 or 19 or 20 or 21 | | |
| 23 | 9 and 22 |  |  |
