# Supplementary material for: Models of Telehealth Service Delivery in Adults With Spinal Cord Injuries: Scoping Review
Source: JMIR Rehabil Assist Technol. 2023 Jun 29;10:e41186. doi: 10.2196/41186 (PMC10365587; doi:10.2196/41186)
Supplement: Multimedia Appendix 3 [file rehab_v10i1e41186_app3.docx]

**Scoping Review – Telehealth for Community-Based Sample of Persons with Spinal Cord Injury (SCI)**

**Data Extraction Form for Quantitative Studies**

| Reviewer’s Initials: | | | | | | | | Date: |
| --- | --- | --- | --- | --- | --- | --- | --- | --- |
| First author’s last name: | | | | | | | | Year: |
| **Eligibility:** | | | | | | | | |
| ☐ PT or ☐ OT or ☐ Both | | |  | | | | ☐ English language | |
| ☐ Age ≥18 yrs. | | |  | | | | ☐ Community-Based persons with SCI | |
| ☐ High or middle income country (Specify): | |  | | | | | ☐ Model of service provision or intervention  ☐Virtual delivery of interventions | |
| **Article type:** | | | | | | | | |
| ☐Non-experimental design | | | ☐Experimental design | | | | | |
| ☐Qualitative study  ☐Editorial  ☐Practice guideline  ☐Review  ☐Other: | | | Interventional study  ☐Pre-post  ☐Non-randomized trial  ☐RCT  ☐Other: | | | | Observational study  ☐Cross sectional  ☐Cohort  ☐Other: | |
| **Participant characteristics:** | | | | | | | | |
| Total no. of participants | | |  | | | |  |  |
| Age | | |  | | | |  |  |
| Sex | | | ☐ male ☐ female | | | |  |  |
| Marital status | | | ☐ Single ☐Married/Common-Law  ☐ Divorced/Separated ☐ Widowed | | | |  |  |
| living arrangements | | | lives ☐ alone ☐ with family ☐with friends ☐ other: | | | |  |  |
| income status | | | ☐ low-income ☐ middle-income ☐ high-income | | | |  |  |
| education level | | | ☐ grade school ☐ high school  ☐ college ☐ university  ☐ graduate school | | | |  |  |
| working/studying status | | | ☐ student full-time  ☐ student part-time  ☐ working full-time  ☐working part-time ☐ unemployed ☐ retired | | | |  |  |
| time since injury | | | ☐ ﹤1 year ☐ 1-2 years  ☐ 3-5 years ☐ 6-10 years  ☐＞10 years | | | |  |  |
| details of SCI | | | ☐ traumatic  ☐ Non-traumatic  ☐ Tetraplegia  ☐ Paraplegia  ☐ Incomplete  ☐ Complete | | | |  |  |
| Mobility | | | ☐ Power wheelchair  ☐ Manual wheelchair  ☐ Ambulatory with ___________ (aid) | | | |  |  |
| Comorbidities with SCI | | | ☐ Arthritis ☐ Hypertension  ☐ Heart disease ☐ Stroke  ☐ Diabetes ☐ COPD  ☐ Cancer ☐ Mental illness  ☐ Other (specify): | | | |  |  |
| Secondary complications related to SCI | | | ☐ Pressure sores ☐ Muscle spasms  ☐ Bladder/bowel dysfunction  ☐ Urinary infection  ☐ Chronic pain  ☐ Sexual dysfunction  ☐ Joint and muscle pain  ☐ range of psychological problems  ☐ Otheris (specify): | | | |  |  |
| **Article details:** | | | | | | | | |
| Population: ☒ Spinal Cord Injury Population | | | | | | | | |
|  | | | | | | | | |
| Setting | ☐Community (Specify): | | | | | | | |
|  | ☐Transitional care | | | | |  | | |
| **Research Question/Purpose:** | | | | | | | | |
|  | | | | | | | | |
| **The paper describes an ☐ Intervention or ☐ Model of service delivery** | | | | | | | | |
| Theoretical Framework or Model Yes ☐ No ☐ Details: | | | | | | | | |
| **Intervention details:** | | | | | | | | |
| ☐ Chronic disease management/self-management | | | | | ☐ Exercise program/prescription | | | |
| ☐ Pain management/self-management | | | | | ☐ Mobility aid/equipment prescription | | | |
| ☐ Return to work | | | | | ☐ Other: | | | |
| ☐ ADL training | | | | |  | | | |
| Format/Delivery | | | | Delivered by | | | | Intensity, frequency, duration |
| ☐ Video-conferencing software (video-telehealth)  ☐ remote/tele- web-based applications/programs/ platforms that include different contents (i.e., modules, homework, educational videos/images, and email/text/phone support from the provider/facilitator)  ☐ Telephone calls  ☐ Other: | | | |  | | | |  |

| **Additional details about the intervention** | | | | |
| --- | --- | --- | --- | --- |
|  | | | | |
| **Model of Service Delivery details:** | | | | |
|  | | | | |
| **Describes the role of the OT/PT ☐ Yes ☐ No Please provide details.** | | | | |
|  | | | | |
| **Describes OT/PT needs for the spinal cord injury population ? ☐ Yes ☐ No** | | | | |
| ☐Physical health problem  ☐Mental health problem  ☐Navigating community resources/programs  ☐ General health/lifestyle issue | | | ☐Referral to other health care providers  ☐ Referral to other sectors (Social services)  ☐Other: | |
| **Describes facilitators and barriers to service delivery in spinal cord injury populations? ☐ Yes ☐ No** | | | | |
| Barriers: | | | Facilitators: | |
|  | | |  | |
| **Describes health outcomes used in spinal cord injury populations? ☐ Yes ☐ No Please list.** | | | | |
|  | | | | |
| **For experimental studies only** | | | | |
| Sample Size | Total | Int: | | Control: |
| Groups’ mean age | | Int: | | Control: |
| Sex | | Int: %F %M %Other | | Control: %F %M %Other |
| Results: | | | | |
|  | | | | |
| **Study limitations** | | | | |
|  | | | | |

**Data Extraction Form for Qualitative or Mixed Methods Studies**

| Reviewer’s Name: shaghayegh mirbaha | | Date: |
| --- | --- | --- |
| First author’s last name: | | Year: |
| Type of study | ☐ Qualitative ☐ Mixed Methods ☐Editorial ☐Practice guideline ☐Review ☐Other: | |
| Research approach | ☐ Phenomenology ☐ Ethnography ☐ Grounded Theory ☐ Other | |

| Article details |
| --- |
| Population: ☒ Spinal Cord Injury Population |

| Participant Characteristics | |
| --- | --- |
| Total no. of participants |  |
| Age |  |
| Sex | ☐ male ☐ female |
| Marital status | ☐ Single ☐ Married/Common-Law ☐ Divorced/Separated ☐ Widowed |
| living arrangements | lives ☐ alone ☐ with family ☐ with friends ☐ other: |
| income status | ☐ low-income ☐ middle-income ☐ high-income |
| education level | ☐ grade school ☐ high school ☐ college ☐ university  ☐ graduate school |
| working/studying status | ☐ student full-time ☐ student part-time ☐ working full-time ☐working part-time ☐ unemployed ☐ retired |
| time since injury | ☐ ﹤1 year ☐ 1-2 years ☐ 3-5 years ☐ 6-10 years ☐＞10 years |
| details of SCI | ☐ traumatic ☐ Non-traumatic  ☐ Tetraplegia ☐ Complete ☐ Paraplegia ☐ Incomplete |
| Mobility | ☐ Power wheelchair ☐ Ambulatory with ___________ (aid)  ☐ Manual wheelchair |
| Comorbidities with SCI | ☐ Arthritis ☐ Hypertension ☐ Heart disease ☐ Stroke ☐ Diabetes  ☐ COPD ☐ Cancer ☐ Mental illness ☐ Other (specify): |
| Secondary complications related to SCI | ☐ Pressure sores ☐ Muscle spasms ☐ Bladder/bowel dysfunction  ☐ Urinary infection ☐ Chronic pain ☐ Joint and muscle pain  ☐ range of psychological problems ☐ Sexual dysfunction  ☐ Otheris (specify): |

| Methods & Results |
| --- |

| Study purpose |  | | |
| --- | --- | --- | --- |
| Design | ☐ Interviews | | ☐ Observations |
|  | ☐ Questionnaires | | ☐ Focus groups |
|  | ☐ Surveys | | ☐ Other: |
| Setting |  | | |
| Duration of participation |  | | |
| Main Findings/Results |  | | |
| Conclusions |  | | |
| limitations & future directions |  | | |
| Ethical approval obtained for study | ☐ Yes ☐ No ☐ Unclear | Withdrawals from study: | |
